# A Label-Free Electrochemical Aptasensor Based on Zn/Fe Bimetallic MOF Derived Nanoporous Carbon for Ultra-Sensitive and Selective Determination of Paraquat in Vegetables

**DOI:** 10.3390/foods11162405

**Published:** 2022-08-10

**Authors:** Qiaoling Wu, Han Tao, Yuangen Wu, Xiao Wang, Qili Shi, Donglin Xiang

**Affiliations:** 1 School of Liquor and Food Engineering, Guizhou University, Guiyang 550025, China; 2 Key Laboratory of Fermentation Engineering and Biopharmacy of Guizhou Province, Guizhou University, Guiyang 550025, China

**Keywords:** paraquat, Zn/Fe bimetallic MOF, nanoporous carbon, electrochemical aptasensor, label-free, food safety

## Abstract

Paraquat (PQ) has high acute toxicity, even at low concentrations. For most people, the main pathway of exposure to PQ is through the diet. Therefore, the development of simple and efficient methods for PQ testing is critical for ensuring food safety. In this study, a new electrochemical detection strategy for paraquat is proposed based on the specific binding of PQ to its nucleic acid aptamer. Firstly, the Zn/Fe bimetallic ZIF derived nanoporous carbon (Zn/Fe-ZIF-NPC) and nickel hexacyanoferrate nanoparticles (NiHCF-NPs) were sequentially modified onto the glassy carbon electrode (GCE). NiHCF-NPs served as the signal probes, while Zn/Fe-ZIF-NPC facilitated electron transfer and effectively enhanced the sensing signal of NiHCF-NPs. Au nanoparticles (AuNPs) were then electrodeposited on the NiHCF-NPs/Zn/Fe-ZIF-NPC/GCE and then the thiolated aptamer was assembled on the AuNPs/NiHCF-NPs/Zn/Fe-ZIF-NPC/GCE via Au-S bonding. When incubated with PQ, the formation of PQ–aptamer complexes delayed the interfacial electron transport reaction of NiHCF-NPs, which caused a decrease in the current signals. As a result, simple and highly sensitive detection of PQ can be readily achieved by detecting the signal changes. A linear range was obtained from 0.001 to 100 mg/L with a detection limit as low as 0.34 μg/L. Due to the recognition specificity of the aptamer to its target molecule, the proposed method has excellent anti-interference ability. The prepared electrochemical aptasensor was successfully used for PQ assay in lettuce, cabbage and agriculture irrigation water samples with recoveries ranging from 96.20% to 104.02%, demonstrating the validity and practicality of the proposed method for PQ detection in real samples.

## 1. Introduction

Food safety has always been a global issue of great concern. Among the various food hazards, the adverse effects caused by excessive pesticide residues in food cannot be neglected. The abuse of pesticides not only causes environmental pollution, but also poses a serious threat to public health.

Paraquat (PQ), 1,1-dimethyl-4,4-bipyridine dichloride, is one of the most widely used herbicides [1] in the world because of its low cost and nonselective features. However, paraquat is highly toxic and can cause severe damage to vital organs such as the lung and liver [2,3], as well as inducing cancer and weakening immunity [4]. As a result, the EU has banned PQ since 2007 and has listed PQ in the database of compounds that should be monitored in food samples [5]. China has also banned the use of PQ since July 2016, but illegal use of paraquat still occurs frequently and therefore the maximum residue limits (MRLs) for PQ in different foods are still regulated in the latest Chinese food safety standards (GB 2763-2021). Moreover, more than 90 countries worldwide still allow the use or limited use of PQ [6,7,8]. However, due to its chemical stability and solubility in water, PQ can easily accumulate in the environment and in agricultural products, and then enter the human body via food chain. PQ contamination in agricultural products is one of the highly concerning food safety issues in many countries [9]. It has been reported that PQ was detected in various food products including cabbage, guava, eggplant and round gourd [10,11]. Therefore, the development of rapid and accurate PQ detection methods is essential for the timely protection of consumer health.

In the past, efforts and achievements have been made for PQ detection by employing Liquid Chromatography (LC) [12], Gas Chromatography-Mass Spectrometry (GC-MS) [13], Surface enhanced Raman spectroscopy (SERS) [14], etc. However, expensive instruments, time-consuming pre-processing steps and specialized operations do not allow for the low cost, simple and fast detection of PQ, so new detection methods still need to be developed. Electroanalysis technology has become a promising food safety testing technique due to its low cost, high sensitivity and simple operation [15,16]. For PQ assay, the currently reported electrochemical method is basically based on direct reduction of PQ [17,18]. However, when the sample matrix is complex, coexisting species which can also be reduced near the PQ reduction potential will seriously interfere with PQ determination, i.e., there is a deficiency of low detection specificity. 

Aptamers are monomeric DNA or RNA oligonucleotide molecules that are chemically synthesized in vitro and can bind target molecules with high specificity [19,20], and have been widely used in biosensors as a new generation of biometric molecules [21]. Inspired by this, we considered that the combination of PQ aptamer with electrochemical sensing could achieve high specificity detection of PQ. It is exciting that the PQ aptamer has been successfully obtained by our group with the Capture-SELEX technique [22].

To achieve high sensitivity PQ detection simultaneously, the electrochemical sensing signal needs to be effectively improved, and the simplest way is to modify the electrodes with functional materials. Metal organic framework (MOF) materials, a new category of nanoporous substances, are crystalline porous coordination polymers formed by assembling metal ions or metal clusters with organic ligands [23]. The organic ligands in MOFs contain a large amount of carbon, thus the nanoporous carbon (NPC) materials can be obtained by direct carbonization of MOFs. In contrast to inorganic porous materials (e.g., molecular sieves), NPC has a large specific surface area, rich pore architecture, high stability and electrical conductivity, which can effectively promote electron transfer [24], and is often used as a heterogeneous catalyst to improve the catalytic activity of different electrode reactions [25]. Moreover, NPC also has good biocompatibility [26]. Therefore, it can be used as an ideal material for modifying electrodes with a view to improving the performance of the electrodes.

Herein, we propose a new label-free electrochemical aptasensor that can be used for the determination of PQ in vegetables with high specificity and sensitivity. The sensing interface was designed using NiHCF-NPs, immobilized directly on the working electrode as redox probes to produce sensing signals, thus overcoming the disadvantage of needing to label electroactive substances on the aptamer or target molecule. Zn/Fe-ZIF-NPC modified on GCE acted as an electrocatalyst to improve the electrochemical signal produced by NiHCF-NPs. Subsequently, AuNPs were electrodeposited on the NiHCF-NPs/Zn/Fe-ZIF-NPC/GCE. The deposited AuNPs not only bound the aptamer to the GCE surface, but also greatly increased the sensing signals. After the PQ molecules in test solution were bound to the Apt/AuNPs/NiHCF-NPs/Zn/Fe-ZIF-NPC/GCE via the affinity of aptamer, the change of current signal was measured and used as the quantitative indicator to achieve a simple, reliable and highly sensitive detection of PQ. To the best of our knowledge, although three aptamer-based PQ detection methods have been reported [22,27,28], they are all based on the colorimetric technique. So far, electrochemical aptasensors for PQ detection have not been reported yet.

## 2. Experimental Section

### 2.1. Apparatus and Reagents

Apparatus: Scanning electron microscopy (SEM) observations were made with SUPRA 40 microscope (ZEISS, Germany). N_2_ adsorption/desorption measurements (77.3 K) were performed using the Micromeritics ASAP2020 sorptometer (Micromeritics, USA). Atomic Force Microscopy (AFM) observation was done with a Dimension FastScan (Bruker, Germany). Electrochemical experiments were conducted on a CHI660e electrochemical workstation (CH Instrument Company, China). A GCE (3 mm diameter) was taken as working electrode, while an Ag/AgCl electrode and a Pt electrode were taken as reference and counter electrode, respectively.

Reagents: Zn(NO_3_)_2_·6H_2_O, FeSO_4_·7H_2_O, NH_4_OH, 2-Methylimidazole, K_3_[Fe(CN)_6_], K_4_[Fe(CN)_6_], HAuCl_4_, PQ, Tris-HCl buffer, 6-mercapto-1-hexanol (MCH), Tris(2-carboxyethyl)phosphine Hydrochloride(TCEP), methanol and ethanol were obtained from Aladdin Chemical (Shanghai, China). KCl, NiCl_2_, Na_2_HPO_4_, NaH_2_PO_4_ were purchased from Macklin reagent corporation (Shanghai, China). The thiolated PQ-15 sequence was synthesized by Sangon Biotech. Co., Ltd. (Shanghai, China): 5′-sh-(CH_2_)_6_-ACC GAC CGT GCT GGA CTC TCG CGC AGC GCC GAG TCC GAA CTG GGT GGG GAG TAT GAG CGA GCG TTG CG-3′.

### 2.2. Screening for PQ Aptamer

Aptamer of PQ (PQ-15) was screened in vitro by Capture-SELEX technique according to literature [22]. The biotin-labeled auxiliary primer P3 (5′-Biotin-CGCAACGCTCGC-3′) was paired with the 3′-end of a random single-stranded oligonucleotide library (5′-ACCGACCGTGCTGGACTCT-N30-AGTATGAGCGAGCGTTGCG-3′) by base complementing, and subsequently immobilized on streptavidin-modified magnetic beads. Then, different concentrations of PQ solutions (500–50 μM) were incubated with magnetic beads and the ssDNA with PQ specific affinity was obtained after elution, which was subjected to asymmetric PCR amplification and purification of the product to obtain a secondary ssDNA library for the next round of screening. After 9 rounds of SELEX screening, the collected PQ affinity sequences were cloned for sequencing, then 15 ssDNA sequences with PQ affinity were obtained. The 15 ssDNA sequences were subjected to homology analysis and secondary structure prediction, and then 6 ssDNA sequences were screened and their dissociation constants (K_d_) were calculated, and finally the PQ-15 sequence with the highest affinity for paraquat was selected.

### 2.3. Synthesis of Zn/Fe-ZIF-NPC

According to previous literature [29], Zn/Fe bimetallic zeolite type MOF (Zn/Fe-ZIF) was first synthesized as follows: The solution A was obtained by weighing 21.27 g of Zn(NO_3_)_2_·6H_2_O and 0.994 g of FeSO_4_·7H_2_O into a flask containing 100 mL of methanol and stirring continuously until completely dissolved. Solution B was obtained by adding 3.75 g of 2-methylimidazole to 100 mL of methanol and stirring until completely dissolved. Solution A and solution B were mixed and stirred to react at room temperature for 24 h. Following centrifugation at 10,000 rpm for 10 min, the sediment was washed several times with methanol and subsequently dried at 50 °C under vacuum to give Zn/Fe-ZIF. Next, 1 g of Zn/Fe-ZIF was added into the mixture of 10 mL furfuryl alcohol and 1 mL ammonium hydroxide and stirred overnight. The product was then washed, dried and transferred to a tube furnace and heated at 80 °C under N_2_ protection for 24 h, then held at 150 °C for 6 h and finally heated to 900 °C at 2 °C min^−1^ for 2 h. The resulting solid was washed with ethanol and ultrapure water and freeze-dried to give Zn/Fe-ZIF-NPC. To modify GCE, Zn/Fe-ZIF-NPC dispersion (1 mg/mL) was prepared by dispersing 2 mg of Zn/Fe-ZIF-NPC in 2 mL of water/isopropyl alcohol/0.1 wt% nafion (volume ratio 7.8:2.0:0.2). Zn/Fe-ZIF dispersion (1 mg/mL) was obtained in the same way.

### 2.4. Synthesis of NiHCF-NPs

NiHCF-NPs were obtained according to literature [30]. At first, 70 mL of 0.01 mol/L NiCl_2_ solution was added drop by drop into 70 mL of 0.05 mol/L K_3_[Fe(CN)_6_] solution containing 0.05 mol/L KCl, and vigorous stirring was continued for 5 min. The solution was then immediately centrifuged and the solid fraction was collected and washed three times with ultrapure water. Finally, the solids were vacuum dried at 40 °C for 12 h to give yellow NiHCF-NPs. NiHCF-NPs were dispersed in water to produce a 1 mg/mL dispersion, which was stored at 4 °C prior to use.

### 2.5. Fabrication of Aptasensor

Briefly, the GCE surface was polished with Al_2_O_3_ power (0.05 μm) and ultrasonically cleaned with ethanol and water. Then, 8 μL of Zn/Fe-ZIF-NPC solution was dropped on GCE surface and dried at room temperature. Subsequently, 6 μL of NiHCF-NPs solution was coated on the Zn/Fe-ZIF-NPC/GCE. After thorough drying, the NiHCF-NPs/Zn/Fe-ZIF-NPC/GCE were placed in 1 mM HAuCl_4_ containing 0.1 M KCl and 50 mM H_2_SO_4_, where AuNPs were electrodeposited on the NiHCF-NPs/Zn/Fe-ZIF-NPC/GCE by 12 cyclic voltametric scans over 0∼+1.0 V range at a scan rate of 50 mV s^−1^. The modified GCE was denoted as AuNPs/NiHCF-NPs/Zn/Fe-ZIF-NPC/GCE.

**Scheme 1:**
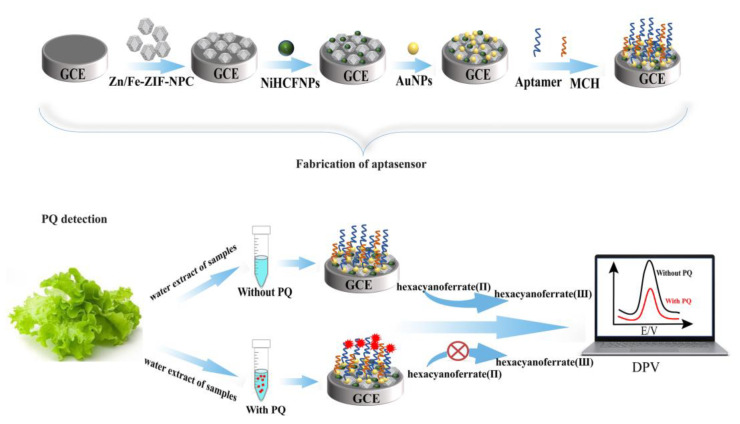
Illustration of the aptasensor fabrication process and PQ detection.

Prior to immobilization, 20 μM of aptamer was mixed with 2 mM TCEP to reduce disulfide bonds in the aptamer, and then heated at 95 °C for 5 min with a constant temperature metal bath. Next, it was placed in a refrigerator at 4 °C for 15 min. The aptamer concentration was then diluted to 2.5 μM with 20 mM Tris-HCl buffer (pH 7.0). Subsequently, the AuNPs/NiHCF-NPs/Zn/Fe-ZIF-NPC/GCE was incubated with 2.5 μM of aptamer at 4 °C for 12 h to bind the aptamer to the working electrode via Au-S bonds. Finally, 6 μL of 1 mM MCH was placed on the modified working electrode to block non-specific adsorption at room temperature for 40 min and the resulting working electrode was labelled as MCH/Apt/AuNPs/NiHCF-NPs/Zn/Fe-ZIF-NPC/GCE. The fabrication procedure is shown in Scheme 1.

### 2.6. Electrochemical Measurement and PQ Detection

Cyclic voltammetry (CV) and electrochemical impedance spectroscopy (EIS) were used to characterize the preparation process of the aptasensor. CV experiments were performed in 0.1 M PBS (pH 7.0) form −0.1∼0.8 V at the scan rate of 50 mV s^−1^. EIS experiments were performed in 0.1 M KCl solution containing a 5 mM [Fe(CN)_6_]^3−/4−^(1:1) mixture with a voltage frequency range of 0.01 Hz to 10 kHz, and the electron transfer resistance (*R_et_*) was calculated based on Randles equivalent circuit to evaluate the electron transfer ability of different modified GCEs. For PQ detection, aptasensors were incubated with test solutions containing different concentrations of PQ for 60 min, washed carefully with 0.1 M PBS and then immersed in 0.1 M PBS (pH 7.0) to record the current responses by differential pulse voltammetry (DPV). The percentage drop in current signal (*ΦI*) was calculated according to the following formula:

$$\Phi I\left(\%\right)=\left(I_{0}-I_{1}\right)/I_{0}\times 100$$

where *I*_0_ and *I*_1_ represent the peak current before and after incubation with PQ, respectively. In DPV experiments, the pulse amplitude was 0.05 V, the pulse width was 0.05 s and the pulse period was 0.5 s. The stability, reproducibility and interference tests of the sensors were also investigated by DPV.

### 2.7. Real Samples Preparation

Vegetables and river water (irrigation water) located at vegetable growing areas were selected as actual samples. Cabbage and lettuce samples were obtained from farmers in Huaxi District, Guiyang City, China. Sample preparation was conducted referring to previous literature [31]. Fresh lettuce and cabbage were cut into small pieces, respectively. Then, 20 g samples were homogenized with 50 mL water in a stirrer for 5 min and the mixture was then sonicated for 1 h. After filtration through a 0.22 μm membrane, the filtrate collected was used as the test samples. Water samples were taken from the Huaxi River (26°44′36″ N, 106°67′82″ E), which is used as irrigation water for farmland. Prior to testing, the water sample was first centrifuged to remove solid particles, and then the supernatant was filtered through a 0.22 µm membrane and used as the test sample directly. For recovery experiments, the above test samples were spiked with PQ standard solution to configure samples with different PQ concentrations.

## 3. Results

### 3.1. Characterization of Nanomaterials

**Figure 1:**
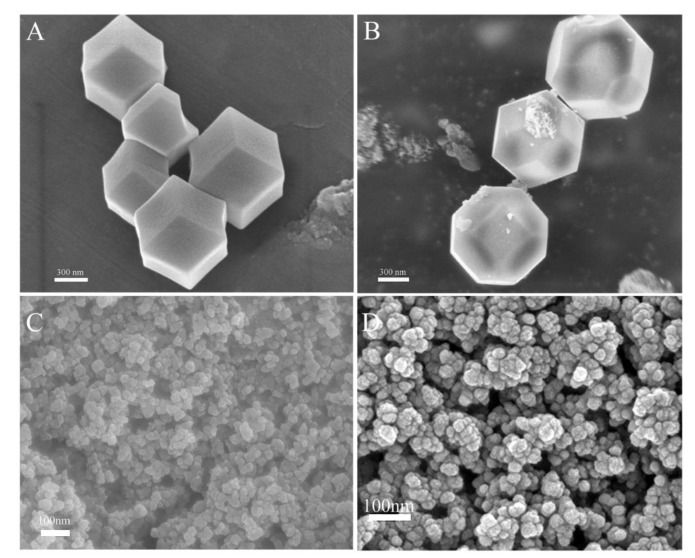
SEM images of Zn/Fe-ZIF (**A**), Zn/Fe-ZIF-NPC (**B**), NiHCF-NPs (**C**) and AuNPs (**D**).

The surface morphology of Zn/Fe-ZIF and Zn/Fe-ZIF-NPC were characterized using SEM. Figure 1A shows Zn/Fe-ZIF has a rhombic dodecahedral structure with a diameter of approximately 700 nm. After carbonization, the obtained Zn/Fe-ZIF-NPC still retains the rhombic dodecahedral structure of Zn/Fe-ZIF precursor (Figure 1B). The morphology of NiHCF-NPs was shown in Figure 1C. It can be seen that the prepared NiHCF-NPs have a diameter of about 20–40 nm. Figure 1D is the SEM image of electrodeposited AuNPs. It can be observed that the gold particles are evenly distributed with a diameter of about 30 nm.

**Figure 2:**
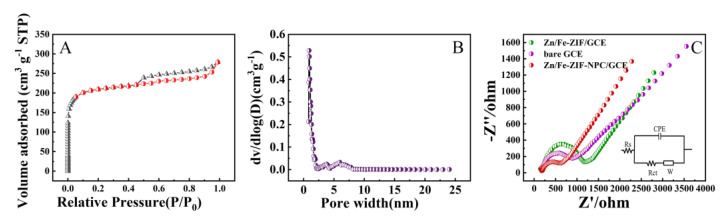
(**A**) N_2_ adsorption/desorption isotherms of Zn/Fe-ZIF-NPC, (**B**) Pore size distributions of Zn/Fe-ZIF-NPC, (**C**) EIS of bare GCE, Zn/Fe-ZIF/GCE and Zn/Fe-ZIF-NPC/GCE in 0.1 M KCl (containing 5 mM [Fe(CN)_6_]^3−/4−^). Zn/Fe-ZIF loading volume: 8 μL; Zn/Fe-ZIF-NPC loading volume: 8 μL.

The specific surface area and pore size distribution of Zn/Fe-ZIF-NPC were investigated by nitrogen adsorption/desorption experiments carried out at 77.3 K. As displayed in Figure 2A, the nitrogen adsorption/desorption isotherms of Zn/Fe-ZIF-NPC were type IV with a conspicuous H2-type hysteresis loop, indicating that Zn/Fe-ZIF-NPC has high porosity. The pore size distribution curves (Figure 2B) illustrate that the Zn/Fe-ZIF-NPC possesses a hierarchical porous structure with micropores and mesopores. Besides, the Brunauer–Emmett–Teller (BET) surface area of Zn/Fe-ZIF-NPC was calculated as 401.66 m^2^ g^−1^. The high specific surface area and porosity make Zn/Fe-ZIF-NPC possess good absorbability, which is conducive to the immobilization of NiHCF-NPs redox probe.

Besides, electrochemical AC impedance spectroscopy were recorded to compare the interface properties of Zn/Fe-ZIF/GCE, Zn/Fe-ZIF-NPC/GCE and bare GCE in 0.1 M KCl solution containing 5 mM [Fe(CN)_6_]^3−/4−^(1:1). Fitted the Nyquist plots in Figure 2C with Randles Equivalent circuit, the electron transfer resistance (*R_et_*) for different GCEs was: Zn/Fe-ZIF/GCE (1245 Ω) > bare GCE (692 Ω) > Zn/Fe-ZIF-NPC/GCE (445 Ω). Compared with Zn/Fe-ZIF/GCE and bare GCE, Zn/Fe-ZIF-NPC/GCE has lower electron transfer resistance, indicating that Zn/Fe-ZIF-NPC can effectively accelerate the electron transfer at the electrode interface, which is undoubtedly beneficial for improving the sensing performance of the aptasensor.

### 3.2. Characterization of Modified GCEs

**Figure 3:**
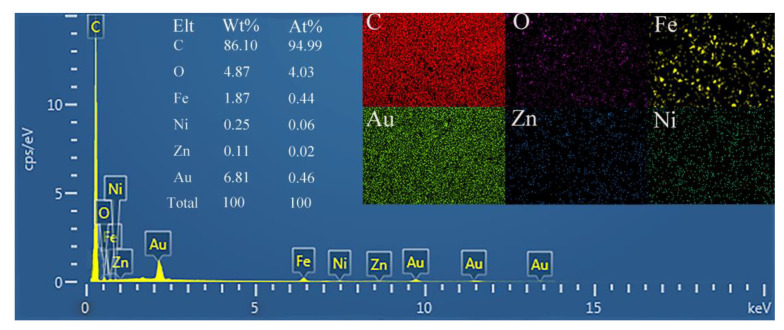
EDS spectrum of AuNPs/NiHCF-NPs/Zn/Fe-ZIF-NPC/GCE. Zn/Fe-ZIF loading volume: 8μL; Zn/Fe-ZIF-NPC loading volume: 8 μL. CV cycle number of AuNPs electrodeposition: 10.

The elemental composition of the AuNPs/NiHCF-NPs/Zn/Fe-ZIF-NPC/GCE surface was determined by EDS. As displayed in Figure 3, Au, Ni, Fe, Zn, C and O elements were found on the AuNPs/NiHCF-NPs/Zn/Fe-ZIF-NPC/GCE surface, and these six elements were highly dispersed on the modified GCE’s surface, indicating that the modification materials were successfully immobilized on the GCE surface.

**Figure 4:**
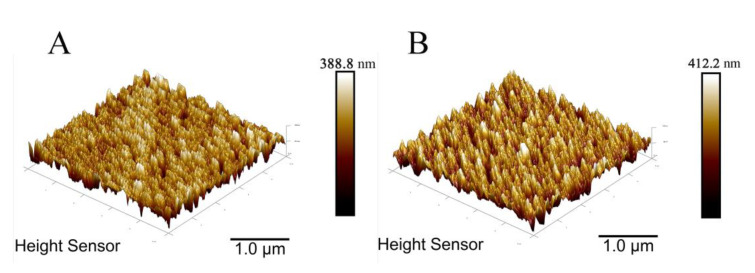
AFM images of AuNPs/NiHCF-NPs/Zn/Fe-ZIF-NPC/GCE (**A**) and Apt/AuNPs/NiHCF-NPs/Zn/Fe-ZIF-NPC/GCE (**B**). Aptamer concentration: 2.0 μM. Other modification conditions are the same as in Figure 3.

The surfaces of AuNPs/NiHCF-NPs/Zn/Fe-ZIF-NPC/GCE and Apt/AuNPs/NiHCF-NPs/Zn/Fe-NPC/GCE were observed by atomic force microscopy (AFM). As shown in Figure 4, the GCE surface became rougher after the bond of aptamer. The surface roughness was calculated from the AFM height images using root-mean-square value (RMS) and surface area difference. The surface height of AuNPs/NiHCF-NPs/Zn/Fe-ZIF-NPC/GCE was obtained as 388.8 nm with an RMS of approximately 33.26 nm, while the surface height of Apt/AuNPs/NiHCF-NPs/Zn/Fe-ZIF-NPC/GCE increased to 412.2 nm and the surface roughness increased to 50.37 nm, which indicates that aptamers were effectively immobilized on the AuNPs/NiHCF-NPs/Zn/Fe-ZIF-NPC/GCE surface.

**Figure 5:**
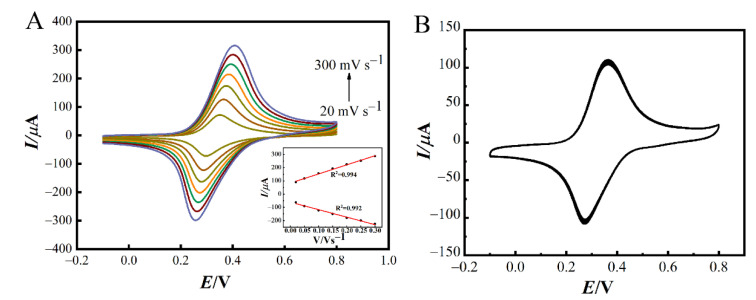
(**A**) CV curves of AuNPs/NiHCF-NPs/Zn/Fe-ZIF-NPC/GCE in 0.1 M PBS at various scan rates (from the inside to out: 0.02, 0.05, 0.10, 0.15, 0.20, 0.25 and 0.30 V s^−1^), (Inset: the relationship between the peak current and scan rate), (**B**) CV curves of AuNPs/NiHCF-NPs/Zn/Fe-ZIF-NPC/GCE in 0.1 M PBS at 0.05 V s^−1^. The modification conditions are the same as in Figure 3.

Moreover, the prepared AuNPs/NiHCF-NPs/Zn/Fe-ZIF-NPC/GCE were characterized using the CV technique. Firstly, the CV curves of AuNPs/NiHCF-NPs/Zn/Fe-ZIF-NPC/GCE at different scan rates were recorded. As shown in Figure 5A, higher current signals are obtained at higher scan rates and there is a good linear relationship between peak current and scan rate (inset of Figure 5A), corresponding to equations $I_{pa}\;\left(\mu A\right)=83.41v\;\left(V\;s^{-1}\right)+689.06\left(R^{2}=0.994\right)$ and $I_{pc}\;\left(\mu A\right)=-60.37v\;\left(V\;s^{-1}\right)-568.43\left(R^{2}=0.992\right)$. The above phenomena indicate that the electrochemical reaction that occurred on the GCE was an adsorption-controlled process. Then, the prepared AuNPs/NiHCF-NPs/Zn/Fe-ZIF-NPC/GCE were placed in 0.1 M PBS and 30 consecutive cyclic voltammetric scans were conducted. The results obtained are shown in Figure 5B. It can be noticed that the CV curves were stable and the peak current remained at 93% of the initial value after 30 cycles, demonstrating the good stability of the constructed modified GCEs.

### 3.3. Electrochemical Behaviors of Different GCEs

**Figure 6:**
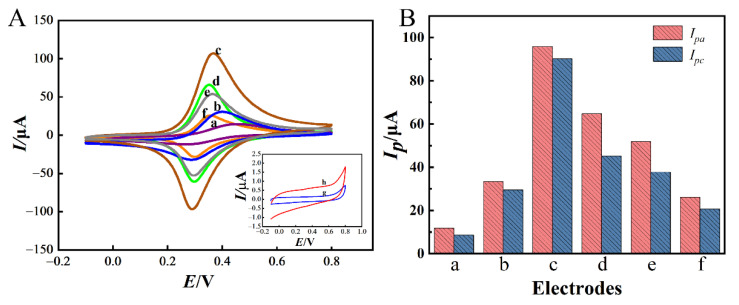
CV curves of different modified GCEs in 0.1 M PBS (pH = 7.0) (**A**) and the peak current values for different modified GCEs (**B**). a: NiHCF-NPs/GCE, b: NiHCF-NPs/Zn/Fe-ZIF-NPC/GCE, c: AuNPs/NiHCF-NPs/Zn/Fe-ZIF-NPC/GCE, d: Apt/AuNPs/NiHCF-NPs/Zn/Fe-ZIF-NPC/GCE, e: MCH/Apt/AuNPs/NiHCF-NPs/Zn/Fe-ZIF-NPC/GCE, f: PQ/MCH/Apt/AuNPs/NiHCF-NPs/Zn/Fe-ZIF-NPC/GCE, g: bare GCE, h: MCH/Apt/AuNPs/Zn/Fe-ZIF-NPC/GCE. The modification conditions are the same as in Figure 4.

Firstly, the electrochemical behavior of different modified GCEs during the aptasensor preparation processes was investigated by CV in 0.1 M PBS (pH = 7.0). As shown in Figure 6A,B, no redox peak appeared on bare GCE (curve g). However, a pair of distinct redox peaks appeared on NiHCF-NPs/GCE (curve a) with the oxidation peak current (*I_pa_*) and reduction peak current (*I_pc_*) of 10.89 μA and 6.65 μA. This pair of redox peaks was caused by the electron transfer between Fe^3+^ and Fe^2+^ in NiHCF-NPs [32]. After modification with Zn/Fe-ZIF-NPC (curve b), the *I_pa_* and *I_pc_* on NiHCF-NPs/Zn/Fe-ZIF-NPC/GCE increased to 33.42 μA and 28.62 μA, respectively, which were significantly higher than those on NiHCF-NPs/GCE, indicating that Zn/Fe-ZIF-NPC effectively enhanced the sensing signal, which was attributed to the promotion of electron transport by Zn/Fe-ZIF-NPC. After electrodeposition on AuNPs (curve c), *I_pa_* and *I_pc_* increased significantly to 95.86 μA and 87.29 μA, indicating that Au also has excellent electrocatalytic activity for the redox of NiHCF-NPs. Compared with NiHCF-NPs/GCE, the *I_pa_* and *I_pc_* signals generated by the NiHCF-NPs probe were 8.8-fold and 13.1-fold greater under the synergistic effect of Zn/Fe-ZIF-NPC and AuNPs, which would greatly improve the sensitivity of PQ detection. When Apt was self-assembled on the modified GCE through Au-S bonding [33], the *I_pa_* and *I_pc_* of Apt/AuNPs/NiHCF-NPs/Zn/Fe-ZIF-NPC/GCE (curve d) decreased to 60.85 μA and 45.18 μA. After blocking the unbound active site on the modified GCE surface with MCH [34], the *I_pa_* and *I_pc_* further decreased (curve e). Finally, after incubation of MCH/Apt/AuNPs/NiHCF-NPs/Zn/Fe-ZIF-NPC/GCE with PQ (10 mg/L) for 60 min, the *I_pa_* and *I_pc_* decreased once again (curve f), which was due to the formation of the PQ-Apt complex on the modified GCE surface impeding the electron transfer at the sensing interface. The above results combined with those in Figure 4 indicate that the aptasensor was successfully fabricated.

**Figure 7:**
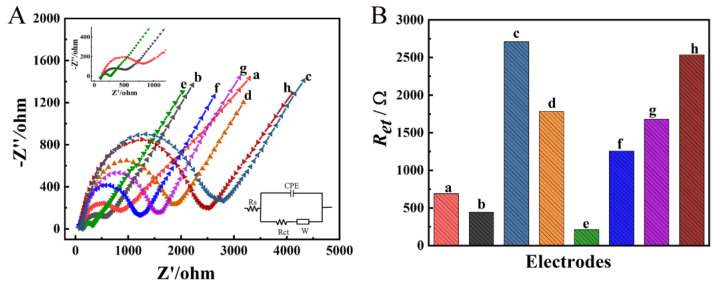
EIS of different modified GCEs in 0.1 M KCl solution containing the 5 mM [Fe(CN)_6_^3−/4−^](1:1) (**A**) and the *Ret* values for different modified GCEs (**B**). a: bare GCE, b: Zn/Fe-ZIF-NPC/GCE, c: NiHCF-NPs/GCE, d: NiHCF-NPs/Zn/Fe-ZIF-NPC/GCE, e: AuNPs/NiHCF-NPs/Zn/Fe-ZIF-NPC/GCE, f: Apt/AuNPs/NiHCF-NPs/Zn/Fe-ZIF-NPC/GCE, g: MCH/Apt/AuNPs/NiHCF-NPs/Zn/Fe-ZIF-NPC/GCE, h: PQ/MCH/Apt/AuNPs/NiHCF-NPs/Zn/Fe-ZIF-NPC/GCE. The modification conditions are the same as in Figure 4.

The interfacial properties of different GCEs were further characterized using EIS in 0.1 M KCl solution containing 5 mM [Fe(CN)_6_]^3−/4−^(1:1). As seen in Figure 7A,B, the *R_et_* of bare GCE was approximately 692 Ω (curve a). After Zn/Fe-ZIF-NPC was modified, the *R_et_* of Zn/Fe-ZIF-NPC/GCE decreased to 445 Ω (curve b). When NiHCF-NPs were further modified on Zn/Fe-ZIF-NPC/GCE, the *R_et_* increased to 1784 Ω because the weak conductivity of NiHCF-NPs severely hindered the electron transfer between the GCE surface and the solution (curve d). However, compared to the *R_et_* of 2712 Ω for NiHCF-NPs/GCE, the *R_et_* of 1784 Ω for NiHCF-NPs/Zn/Fe-ZIF-NPC/GCE confirmed that Zn/Fe-ZIF-NPC can effectively facilitate electron transfer. After electrodeposition of AuNPs, the good conductivity of AuNPs reduced the *R_et_* to 212 Ω (curve e). Subsequently, after aptamer and MCH were attached to the AuNPs/NiHCF-NPs/Zn/Fe-ZIF-NPC/GCE, the *R_et_* increased to 1257 Ω (curve f) and 1680 Ω (curve g) for Apt/AuNPs/NiHCF-NPs/Zn/Fe-ZIF-NPC/GCE and MCH/Apt/AuNPs/NiHCF-NPs/Zn/Fe-ZIF-NPC/GCE, respectively. This sharp increase of *R_et_* can be explained by the electrostatic repulsion between the negatively charged phosphate backbone of Apt and the negative ions of [Fe(CN)_6_]^3−/4−^ [35], and the nonconductive MCH hindered the electron-transfer. Finally, when the target molecule PQ was incorporated, the *Ret* further increased to 2536 Ω (curve h), which was due to the non-conductive PQ-aptamer complex hindering the electron transfer. The results of the EIS experiments were consistent with those of the CV experiments, again confirming the successful construction of the aptasensor. 

### 3.4. Optimization of Experimental Parameters

In order to obtain the best sensing performance, the experimental parameters were optimized using the DPV technique. 

**Figure 8:**
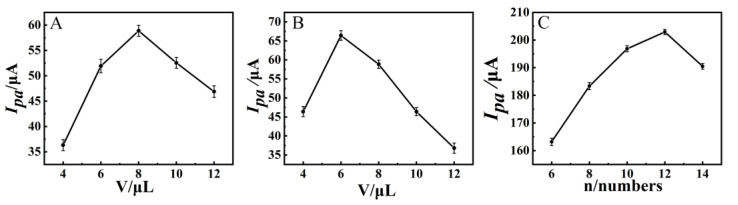
(**A**) Effect of Zn/Fe-ZIF-NPC loading volume on *I_pa_* of NiHCF-NPs/Zn/Fe-ZIF-NPC/GCE, (**B**) Effect of NiHCF-NPs loading volume on *I_pa_* of NiHCF-NPs/Zn/Fe-ZIF-NPC/GCE, (**C**) Effect of CV cycle numbers of AuNPs electrodeposition on the *I_pa_* of AuNPs/NiHCF-NPs/Zn/Fe-ZIF-NPC/GCE. Error bars were obtained as the standard deviation of triple measurements (*n* = 3).

First, the amount of Zn/Fe-ZIF-NPC was optimized (Figure 8A). When the loading volume of Zn/Fe-ZIF-NPC was increased from 4 μL to 8 μL, the current signal increased and reached a maximum at 8 μL, then decreased. This is probably due to the fact that when the loading volume of Zn/Fe-ZIF-NPC was ≤8 μL, a homogeneous film could be formed on the GCE surface and the electron transfer was promoted by Zn/Fe-ZIF-NPC. However, when the loading volume was higher than 8 µL, excessive loading would lead to the aggregation and accumulation of Zn/Fe-ZIF-NPC, which would instead be detrimental to electron transfer. Therefore, 8 μL was chosen as the optimal loading volume for Zn/Fe-ZIF-NPC.

As the signal probe, NiHCF-NPs has a crucial influence on the analytical performance. The impact of NiHCF-NPs amount on the current signal is shown in Figure 8B; with the increase of NiHCF-NPs amount, the current signal first rose and then decreased, and the maximum current signal value was obtained at 6 μL. On the one hand, the more NiHCF-NPs contain more Fe^3+^/Fe^2+^ pairs, which can produce a larger sensing signal. On the other hand, NiHCF-NPs are less conductive, and more NiHCF-NPs impede electron transfer more, which can reduce the sensing signal. The combination of these two aspects resulted in an optimum loading volume of 6 μL.

Furthermore, the amount of AuNPs affects not only the sensing signal, but also the loading amount of aptamer. Within a certain range, the more AuNPs, the greater the sensing signal and the more active sites are available for binding the aptamer. However, too many AuNPs not only do not increase the number of binding sites for the aptamer but also make the sensing signal lower. In the electrodeposition process, the effect of CV scan number on the current signal is shown in Figure 8C. The current signal became larger as the number of cycles increased, and reached the maximum at 12 cycles. Thereafter, the current signal decreased. This is because too many cycles will cause the gold film to be too thick and block the electron transfer. Therefore, the number of CV cycles for depositing AuNPs was chosen to be 12.

**Figure 9:**
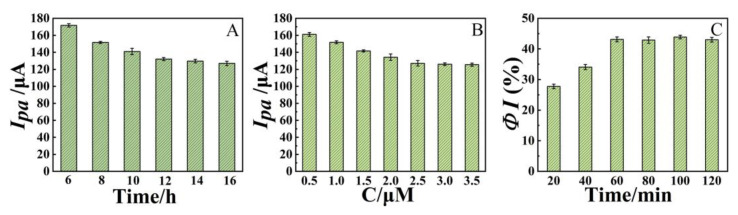
(**A**) Effects of immobilization time of aptamer on *I_pa_*, (**B**) Effects of aptamer concentration on *I_pa_*, (**C**) Effects of PQ incubation time on *ΦI*. Error bars were obtained as the standard deviation of triple measurements (*n* = 3).

The immobilization time and concentration of the aptamer were optimized and the results are presented in Figure 9. It can be seen that the *Ip* decreased with increasing immobilization time and remained almost constant after 12 h (Figure 9A). Hence, the immobilization time of 12 h was selected. In the range of 0.5∼3.5 μmol L^−1^, the *I_pa_* also decreased with increasing concentration and tended to be stable when the concentration exceeded 2.5 μmol L^−1^ (Figure 9B), indicating that the binding of aptamer on the modified GCE surface reached saturation. Therefore, an aptamer of 2.5 μmol L^−1^ was selected for the preparation of the sensor. In addition, the effect of incubation time of PQ (10 mg/L)on the sensing performance was also investigated using percentage drop in current signal (*ΦI*) after incubation as the indicator (Figure 9C). It can be seen that as the incubation time was increased from 20 to 60 min, *ΦI* increased, indicating that more PQ was bound to the modified GCE. After 60 min of incubation, *ΦI* was almost unchanged, indicating that the binding between PQ and aptamer reached equilibrium. Therefore, the incubation time was chosen to be 60 min.

### 3.5. Analytical Performance of the Proposed Aptasensor

**Figure 10:**
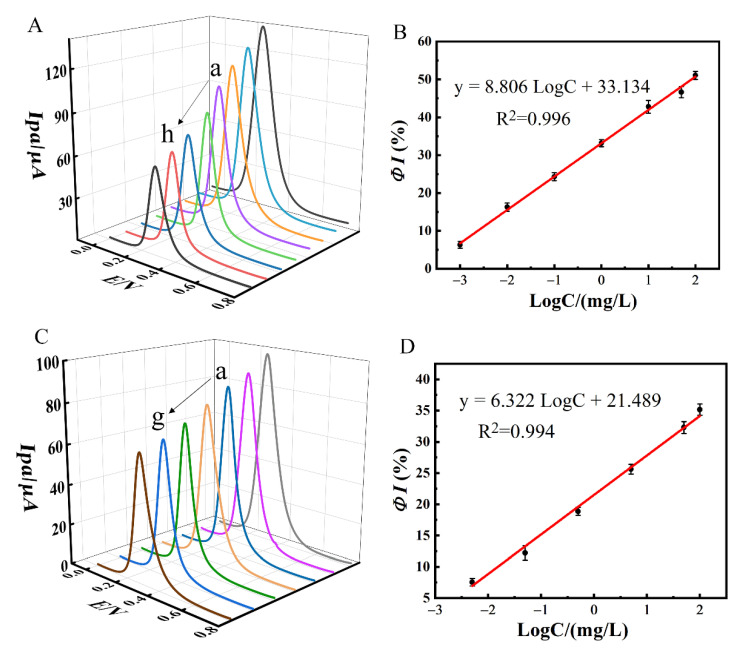
(**A**) DPV responses of MCH/Apt/AuNPs/NiHCF-NPs/Zn/Fe-ZIF-NPC/GCE in 0.1 M PBS at pH 7.0 after incubation with different concentrations of PQ (from a to h: 0, 0.001, 0.01, 0.1, 1.0, 10, 50, 100 mg/L), (**B**) the calibration curve for PQ detecting on MCH/Apt/AuNPs/NiHCF-NPs/Zn/Fe-ZIF-NPC/GCE. (**C**) DPV responses of MCH/Apt/AuNPs/NiHCF-NPs/GCE after incubation with different concentrations of PQ (from a to g: 0, 0.005, 0.05, 0.5, 5.0, 50, 100 mg/L), (**D**) the calibration curve for PQ detecting on MCH/Apt/AuNPs/NiHCF-NPs/GCE. Error bars were obtained as the standard deviation of triple measurements (*n* = 3).

DPV experiments were carried out to assess the analytical performance of the proposed aptasensor towards PQ. The DPV curves obtained are displayed in Figure 10A. It was observed that the current response of DPV gradually decreased with an increase in PQ concentration, which was attributed to the hindrance of electron transfer caused by PQ-aptamer complex formed on the aptasensor surface. Within the range of 0.001 mg/L to 100 mg/L, the *ΦI* versus *log C*_paraquat_ calibration plot is shown in Figure 10B. The regression equation was *ΦI* (%) = 8.806 × logC + 33.134 (R^2^ = 0.996). The LOD was estimated to be 0.34 μg/L based on LOD = 3*S_b_*/*n*, where *S_b_* is the SD of the blank solution and *n* is the slope of the calibration plot.

As a comparison, the aptasensor without Zn/Fe-ZIF-NPC modification was also applied to detect PQ and the results are shown in Figure 10C,D. Again, *ΦI* (%) increased with increasing PQ concentration and the regression equation was *ΦI* (%) = 6.322 logC + 21.489 (R^2^ = 0.994) over the range of 0.005–100 mg/L with an LOD of 2.29 μg/L. It can be noticed that compared to MCH/Apt/AuNPs/NiHCF-NPs/GCE, MCH/Apt/AuNPs/NiHCF-NPs/Zn/Fe-ZIF-NPC/GCE showed higher response sensitivity and lower LOD, which was attributed to the electrocatalytic effect of Zn/Fe-ZIF-NPC on the redox reaction of NiHCF-NPs which effectively improved the sensing signal.

Further, the test performance of the proposed aptasensor was compared with other PQ detection methods including electrochemical or non-electrochemical methods (Table 1). It can be found that the aptasensor proposed in this work has the lowest LOD and wider linear range. Moreover, the proposed aptasensor has the advantages of simplicity, low cost and good stability.

The proposed sensor is more suitable for use in the lab. To achieve in situ detection of PQ, a combination of the proposed method with disposable electrodes (e.g., SPE) is needed, which is the content of our following research.

### 3.6. Reproducibility, Stability and Selectivity

The reproducibility of the proposed aptasensor was assessed. Seven independent sensors were used to detect PQ (10 mg/L) individually. The results showed that *ΦI* was very stable with an RSD of only 2.07%, indicating that the developed aptasensor has good reproducibility.

**Figure 11:**
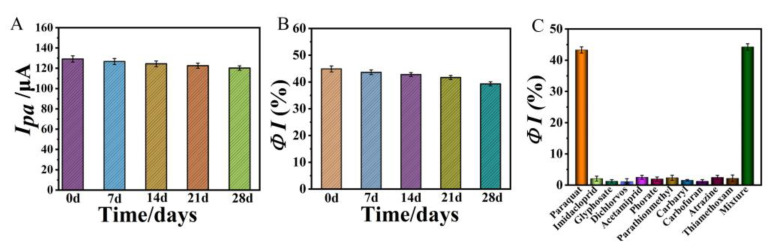
(**A**) Stability of the proposed aptasensor in 0.1 M PBS, (**B**) Stability of the proposed aptasensor for detection of PQ (10 mg/L), (**C**) Selectivity of the proposed aptasensor for PQ detection. Error bars were obtained as the standard deviation of triple measurements (*n* = 3).

Storage stability is an important performance indicator for the sensor. The stability of the prepared aptasensor was examined from two aspects. The same batch of sensors was prepared and stored at 4 °C when not in use. Firstly, three sensors were taken and their current response in 0.1 M PBS was measured periodically. The current response remained at 93% of the initial value after 28 days (Figure 11A), indicating that the modified materials and the NiHCF-NPs probes attached on the modified GCEs were stable during storage. Secondly, at intervals of 7 days, three fresh sensors were taken to measure their detection signal (*ΦI*) for the same concentration of PQ (10 mg/L) and it was observed that the *ΦI* value of the sensors was still 87.71% of the initial value after 28 days (Figure 11B), suggesting that the aptamer still maintained good bioactivity during the storage process. The above experimental results demonstrate good storage stability of the proposed sensor. 

To evaluate the selectivity of the as-prepared aptasensor for PQ detection, a series of pesticides including methomyl, acetamiprid, imidacloprid, dichlorvos, glyphosate, methyl parathion, thiamethoxam, carbofuran, atrazine and carbaryl were selected as interferents. According to the results shown in Figure 11C, there was no significant change in the current response when 10 mg/L of the other pesticides were used instead of 10 mg/L of PQ to incubate with the aptasensor. Meanwhile, when a mixture of 10 mg/L PQ and 10 mg/L other pesticides was incubated with the aptamer sensor, the current response changed significantly and the *ΦI* value was approximately equal to the value of 10 mg/L PQ only, indicating that the as-prepared aptasensor has excellent selectivity for PQ detection, which can be attributed to the specific recognition and affinity effect of the aptamer towards its target molecule.

### 3.7. Detection of PQ in Actual Samples

To verify the viability of the electrochemical aptasensor in practical application, the aptasensor was used for the detection of PQ in lettuce, cabbage and agriculture irrigating water samples. Since PQ was not detected in the original samples, different concentrations of PQ were added into the prepared samples for standard recovery experiments, and each concentration was detected three times in parallel. The recoveries measured were in the range of 96.20% to 104.02% with low RSD as shown in Table 2. This demonstrates the promising application of the constructed electrochemical aptasensor in the detection of PQ.

**Figure 12:**
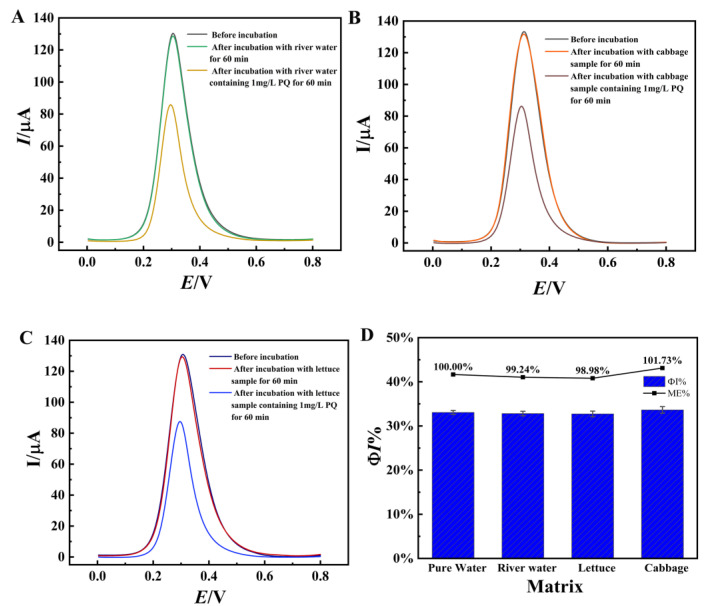
DPV curves of the aptasensors before and after incubation with different actual samples. (**A**) river water sample, (**B**) Cabbage sample, (**C**) lettuce sample and(**D**) *ΦI*% and ME values of the sensors after incubation with actual samples containing 1 mg/L PQ. Error bars were obtained as the standard deviation of triple measurements (*n* = 3).

The possible matrix effects were investigated using the relative response value method and the results are shown in Figure 12. As seen in Figure 12A–C, the proposed sensors showed almost no change in DPV responses after incubation with the original actual samples. Subsequently, after incubation of the sensors with pure water containing 1 mg/L PQ and the actual samples containing 1 mg/L PQ for 60 min, respectively, the current drop percentage (*ΦI* (%)) was measured and the matrix effect was estimated according to ME = A/B, where A is the *ΦI*% caused by PQ in pure water matrix and B is the *ΦI*% caused by the same level of PQ in real sample matrix. It can be seen that the ME was between 98.98–101.73% (Figure 12D). The above results shows that the matrix effect is negligible.

## 4. Conclusions

In this work, a simple label-free electrochemical aptasensor was developed for the selective detection of PQ using NiHCF-NPs as the signal probe. Zn/Fe-ZIF-NPC has large specific surface area and porosity, and effectively accelerated the transfer of electrons. AuNPs also have high electrical conductivity. Based on the dual signal amplification effect of Zn/Fe-ZIF-NPC and AuNPs, highly sensitive detection of ultra-trace PQ was readily achieved with an LOD of 0.34 μg/L and a wide linear range from 0.001 to 100 mg/L. the analysis results of PQ in real vegetable and agriculture irrigating water samples are satisfactory with satisfactory recoveries and low RSD, which proves that the proposed method is highly reliable and practical. Moreover, the sample pre-treatment and electrochemical detection operations of the proposed method are very simple. Therefore, the developed electrochemical aptasensor has the potential to be used as an advanced monitoring tool for the selective and highly sensitive assay of trace PQ residues in vegetable samples.

## Figures and Tables

**Table 1:** Comparison of the proposed aptasensor with other PQ detection methods.

Technique	Methods *	Linear Range (µg L^−1^)	LOD (µg L^−1^)	Ref.
Electrochemistry	Paper-based silver sensor	558.9∼1.863 × 10^4^	149.04	[36]
	Nf/SPGE(gel-based electrolyte platform)	186.3∼1.12 × 10^4^	57.75	[37]
	BN/MoS_2_/AuNPs/GCE	0∼1.863 × 10^4^	13.79	[38]
	LMGCE	9.315∼111.78	0.41	[39]
	CCDs/GCE	18.63∼1863	11.92	[40]
	rGO-AB4	137.86∼1829.47	4.30	[41]
	GC/MoS2/CB [8]-DNPs	136∼1490.4	40.99	[42]
	PtNPs@MIP/SPGrE	9.315∼1.863 × 10^5^	3.73	[31]
Others	fluorescent sensors	5.0∼350	1.20	[7]
	SERS	2.7∼2.7 × 10^4^	2.70	[43]
	Colorimetric detection	9.315∼93.15	4.69	[22]
	Colorimetric detection	0∼5120	267.00	[27]
	Colorimetric detection	60∼200	16.50	[28]
	SALDI-TOF MS	2∼300	0.50	[44]
	HPLC	0.1∼1000	20.00	[45]
	Apt/AuNPs/NiHCF-NPs/Zn/Fe-ZIF-NPC/GCE	1.0∼1.0 × 10^5^	0.34	This work

* NF, nafion; SPGE, screen-printed graphene electrode; BN, boron nitride; GCE, glassy carbon electrode; LMGCE, phospholipid modified electrode; CCDs, carboxylatopillar[5]arene-coated nitrogen-doped carbon dots; rGO, reduced graphene oxide; AB4, activated biochar; GC, Glassy Carbon; CB [8], cucurbit[8]uril; DNPs, diamond nanoparticles; MIP, molecularly imprinted polymer; SPGrE, screen-printed graphene paste electrode; SERS, Surface enhanced Raman spectroscopy; SALDI-TOF MS, Surface-assisted laser desorption/ionization time-of-flight mass spectrometry; HPLC, high-performance liquid-chromatography.

**Table 2:** Detection of PQ in real samples.

Samples	Added (mg/L)	Found (mg/L)	Recoveries (%)	RSD % (*n* = 3)
Water	0.01	0.0104 ± 0.0015	104.02	1.45
	1	0.9852 ± 0.03	98.52	3.32
	10	9.6450 ± 0.18	96.45	1.86
Lettuce	0.01	0.0102 ± 0.0004	102.43	4.35
	1	0.9620 ± 0.027	96.20	2.86
	10	10.3490 ± 0.386	103.49	3.73
Cabbage	0.01	0.0098 ± 0.0004	98.32	3.82
	1	1.0335 ± 0.035	103.35	3.40
	10	9.7790 ± 0.26	97.79	2.72

## Data Availability

The data presented in this study are available on request from the corresponding author.
